# Targeting Skeletal Muscle in Duchenne Muscular Dystrophy: Integrating *in Silico* and Experimental Approaches to Sodium-Glucose Cotransporter-2 Inhibition

**DOI:** 10.1016/j.ajpath.2025.11.002

**Published:** 2025-12-13

**Authors:** Christopher Dostal, Johanna Reiner, Ana I. Antunes Goncalves, Laura S. Sousa, Marlene Knapp, Joel Fischlein, Jessica Marksteiner, Jakob Sauer, Gavin Y. Oudit, Anja Wagner, Dietmar Abraham, Karlheinz Hilber, Klaus Kratochwill, Bruno K. Podesser, Attila Kiss

**Affiliations:** ∗ Center for Biomedical Research and Translational Surgery, Medical University of Vienna, Vienna, Austria; † CBRS Center for Bioinformatic Research and Services e.U., Vienna, Austria; ‡ Ludwig Boltzman Institute for Cardiovascular Research, Vienna, Austria; § Department of Neurophysiology and Pharmacology, Center for Physiology and Pharmacology, Medical University of Vienna, Vienna, Austria; ¶ Division of Cardiology, Department of Medicine, University of Alberta, Edmonton, Alberta, Canada; ‖ Core Facility Proteomics, Medical University of Vienna, Vienna, Austria; ∗∗ Center for Anatomy and Cell Biology, Medical University of Vienna, Vienna, Austria; †† Division of Pediatric Nephrology and Gastroenterology, Department of Pediatrics and Adolescent Medicine, Comprehensive Center for Pediatrics, Medical University of Vienna, Vienna, Austria

## Abstract

Duchenne muscular dystrophy (DMD) is a severe X-linked disorder with progressive myofiber degeneration and fibrosis from dystrophin deficiency. Current therapies are largely supportive with limited anti-fibrotic benefit, prompting new strategies. Sodium-glucose cotransporter-2 inhibitors (SGLT2i) show emerging anti-fibrotic and anti-inflammatory effects. Open-access proteomic and transcriptomic data sets were integrated for *in silico* analyses, including differential gene expression, weighted gene co-expression network analysis, and pathway enrichment, to identify dysregulated pathways potentially reversible by SGLT2i. Immune cell composition was estimated using CIBERSORTx in human and murine data sets. Therapeutic effects were tested with empagliflozin (EMPA) in *mdx* mice (30 mg/kg per day for 4 weeks, starting at 12 weeks) and DMD^*mdx*^ rats (10 mg/kg per day for 4 months, starting at 5 months), with vehicle controls. Validation used quantitative RT-PCR, grip-strength testing, and histologic fibrosis staining. Analyses highlighted dysregulated extracellular matrix organization, cytokine signaling, and immune responses. Forty overlapping genes were identified; hub genes included *COL3A1*, *COL5A2*, and *TGFB1*. EMPA reduced *Tgfb1* expression in DMD rats and significantly decreased collagen deposition in skeletal muscle. Functional testing showed longer grip duration in EMPA-treated mice. Immune profiling revealed shifts in T cells and macrophages, indicating immunomodulation. Findings were consistent across species and data modalities analyzed. These results demonstrate that EMPA modulates fibrosis, inflammation, and muscle endurance in DMD models. These data support repurposing SGLT2i as a promising therapeutic strategy for DMD.

Duchenne muscular dystrophy (DMD) is a severe and progressive genetic disorder caused by mutation(s) in the dystrophin gene, which lead to the absence of dystrophin, a critical protein for muscle integrity.^1^ This disorder primarily affects boys, with an incidence of 1 in 3500 to 5000 male births, resulting in both skeletal and cardiac muscle degeneration, progressive weakness, and premature death, often due to cardiorespiratory failure.^2^ Muscle degeneration is particularly severe in the diaphragm and accessory respiratory muscles, contributing to respiratory complications, whereas the extensive loss of cardiomyocytes predisposes individuals to DMD-associated cardiomyopathy.^3^ The striated skeletal muscle has an intrinsic ability to repair itself following injury, relying on satellite cells and their interaction with immune cells, particularly macrophages, which play a crucial role in regulating muscle repair and extracellular matrix (ECM) remodeling.^4^ However, in DMD, persistent inflammation^5^ and a skewed M1/M2 macrophage balance exacerbate muscle damage and drive fibrosis,^6^ yet these pathways may serve as promising therapeutic targets.^7^

The pathomechanism of DMD is multifactorial, involving oxidative stress,^8^ chronic inflammation,^9^ and mitochondrial dysfunction,^10^ all of which eventually drive skeletal muscle degeneration and cardiac pathology. Currently, there is no curative treatment for DMD. Available therapies focus on slowing disease progression, managing symptoms, and preserving quality of life. Corticosteroids, such as prednisone and deflazacort, are the mainstay of treatment, helping to slow muscle degeneration and delay loss of ambulation; however, long-term use can often lead to adverse effects, such as weight gain and osteoporosis.^11^, ^12^, ^13^, ^14^, ^15^ Therefore, it is urgent to further elucidate the mechanisms underlying muscle dysfunction and to develop strategies to mitigate fibrosis in DMD.

Sodium-glucose cotransporter 2 inhibitors (SGLT2i), a drug class that includes empagliflozin (EMPA), dapagliflozin, and canagliflozin, are primarily used to treat diabetes, heart failure, and chronic kidney disease.^16^^,^^17^ Emerging evidence across the SGLT2i class links their use with preserved skeletal muscle function and reduced tissue fibrosis, prompting interest in potential benefits beyond glycemic control. Proposed mechanisms include increased urinary glucose excretion that promotes lipolysis and ketone body production, providing alternative energy substrates for muscle^18^; attenuation of inflammation and oxidative stress via reductions in hyperglycemia and glucotoxicity to protect contractile tissue^19^^,^^20^; and preservation of mitochondrial function with enhanced ketone utilization that supports ATP production and performance.^21^ In cardiac tissue relevant to DMD-associated cardiomyopathy, EMPA treatment has been reported to rescue abnormally reduced peak Na^+^ current in dystrophin-deficient mouse ventricular cardiomyocytes.^22^^,^^23^ Collectively, these observations suggest that SGLT2i may offer a pharmacologic approach to mitigating muscle dysfunction and fibrosis in DMD.

Mechanistically, SGLT2 inhibition [targeting SGLT2 encoded by solute carrier family 5 member 2 (SLC5A2)] lowers circulating insulin and shifts systemic fuel use toward fatty acids and ketone bodies,^24^ changes that can influence skeletal muscle substrate selection,^25^ oxidation-reduction balance, and mitochondrial workload.^26^ Wild-type muscle and dystrophic mdx muscle differ in metabolic profiles, with mdx tissue often showing altered glycolytic flux,^27^ impaired oxidative phosphorylation,^28^ and heightened oxidative stress relative^29^ to healthy muscle. Given these distinctions, SGLT2i-driven substrate shifts may differentially affect contractile performance, mitochondrial efficiency, and stress responses in dystrophic versus healthy muscle. Accordingly, a working hypothesis is that SGLT2i could exert muscle-type and disease-state–dependent effects, warranting direct evaluation in dystrophic and nondystrophic contexts.

Recent advancements in state-of-the-art transcriptomics and proteomics analysis have significantly enhanced the understanding of the molecular mechanisms underlying DMD. However, comprehensive studies that integrate molecular data to predict the physiological assessments of novel therapeutics remain limited.^30^^,^^31^ Through a computational discovery pipeline, weighted gene co-expression network analysis (WGCNA) emerges as a powerful tool for identifying gene modules associated with specific phenotypes, enabling the pinpointing of critical pathways and gene networks involved in skeletal muscle dysfunction in DMD. This approach is particularly effective in elucidating complex gene interactions and directly linking them to clinical outcomes.^32^^,^^33^ Given the critical role of fibrosis in DMD, the present study investigates how SGLT2 inhibitors, such as EMPA, modulate fibrotic processes and immune responses *in silico*, with the goal of identifying therapeutic targets to mitigate disease progression.

## Materials and Methods

### Acquisition of Public Microarray Data Sets for DMD

A Gene Expression Omnibus (GEO; *https://www.ncbi.nlm.nih.gov/geo*, last accessed February 15, 2025) search was performed using the term Duchenne muscular dystrophy to identify publicly available transcriptomic data sets. Publicly available data sets were obtained for both human and mouse studies. Four human data sets [skeletal muscle control (Ctrl) *n* = 6, DMD *n* = 16, *https://www.ncbi.nlm.nih.gov/geo/query/acc.cgi?acc=GSE38417*; GEO accession number GSE38417; quadriceps muscle, Ctrl *n* = 11, DMD *n* = 12, *https://www.ncbi.nlm.nih.gov/geo/query/acc.cgi?acc=GSE1004*; GEO accession number GSE1004; quadriceps muscle, Ctrl *n* = 14, DMD *n* = 23, *https://www.ncbi.nlm.nih.gov/geo/query/acc.cgi?acc=GSE6011*; GEO accession number GSE6011; and skeletal muscle Ctrl *n* = 13, DMD *n* = 10, *https://www.ncbi.nlm.nih.gov/geo/query/acc.cgi?acc=GSE3307*; GEO accession number GSE3307] and three mouse data sets [calf muscle, Ctrl (C57Bl/6) *n* = 5, DMD *n* = 5, *https://www.ncbi.nlm.nih.gov/geo/query/acc.cgi?acc=GSE72151*; GEO accession number GSE72151; tibialis anterior muscle, Ctrl (C57BL/10SnJ) *n* = 6, DMD *n* = 6, *https://www.ncbi.nlm.nih.gov/geo/query/acc.cgi?acc=GSE7187*; GEO accession number GSE7187; and tibialis anterior muscle, Ctrl (C57BL/10SnJ) *n* = 3, DMD *n* = 4, *https://www.ncbi.nlm.nih.gov/geo/query/acc.cgi?acc=GSE64418*; GEO accession number GSE64418] were selected for substantial analysis. Within human studies, healthy individuals were used as controls.

### Identification of Differentially Expressed Genes

Differential expression analysis was conducted using the GEO2R tool,^34^ an interactive web application provided by GEO for identifying differentially expressed genes (DEGs) in R version 4.2.2 (*https://www.r-project.org*).^35^ GEO2R and the Linear Models for Microarray Data version 3.54.0 package (*https://www.bioconductor.org/packages/release/bioc/html/limma.html*) were used with default settings to reproducibly identify DEGs. This analysis served as the initial step in identifying candidate genes for further investigation. Genes with a *P* < 0.01 (DMD versus Ctrl) were considered significant and further processed.

### Normalization and Standardization of Gene Expression Data

To ensure consistency and comparability across samples, the raw expression data from the human data sets were normalized and log transformed as part of the GEO2R pipeline. For downstream analyses, a standardized quantile normalization was applied across all data sets using the Linear Models for Microarray Data package version 3.54.0 in R version 4.2.2.

### Functional Annotation and Pathway Enrichment of DEGs

Functional enrichment analysis was performed on the significant DEGs using Metascape version 3.5^36^ to elucidate the biological processes and pathways associated with these genes. The analysis within Metascape used multiple databases, including Gene Ontology Biological Processes, Molecular Signatures Database Hallmark Gene Sets, Reactome Gene Sets, Kyoto Encyclopedia of Genes and Genomes Pathway, Wiki Pathways, and Canonical Pathways from Molecular Signatures Database, providing a comprehensive understanding of the biological context of the identified DEGs. The gene list was mapped to unique National Center for Biotechnology Information Entrez Gene identifiers, and the analysis was controlled for multiple testing using the Benjamini-Hochberg method. The top 100 significantly enriched categories were identified and further analyzed, with the top 20 terms visualized to provide insights into the biological functions and pathways associated with the gene set.

### WGCNA and Module Identification

WGCNA was conducted to identify gene modules associated with clinical traits across multiple human data sets. WGCNA was performed separately on four data sets: GSE38417, GSE1004, GSE6011, and GSE3307. This multi–data set approach was chosen to enhance the robustness and generalizability of the findings. Gene expression data from each data set were preprocessed using the PyWGCNA version 2.1.3 package^37^ in Python version 3.10 (*https://www.python.org/downloads*). Modules of co-expressed genes were identified within each data set, with a focus on the positively correlating modules that exhibited the strongest correlation with clinical traits based on *P* values. To ensure consistency, only genes present in at least three of the four positively correlating modules were retained for further analysis. This stringent selection criterion helped to identify key gene modules that are consistently associated with the clinical traits of interest across different data sets.

### Integration of Multi–Data Set Insights via Venn Diagram Analysis

To identify the most relevant genes across different data sets, a Venn diagram analysis was performed. Differential expression (DE) analysis and WGCNA were conducted separately on each of the four human data sets. For the DE analysis and the WGCNA, only genes that overlapped across all four data sets were retained. A Venn diagram was then constructed to explore the overlap between the DE genes and the WGCNA-identified genes, resulting in a final set of 40 genes. This stringent selection process aimed to identify key genes consistently implicated across multiple analyses, warranting further investigation.

### Overrepresentation Analysis of Key Overlapping Genes

An enrichment analysis was performed on the 40 overlapping genes using the Web-Based Gene Set Analysis Toolkit (WebGestalt) version WebGestalt 2024.^38^ The analysis used overrepresentation analysis for *Homo sapiens*, focusing on the Reactome pathway databases.

### Protein-Protein Interaction Network Construction and Hub Gene Prioritization

The 40 overlapping genes identified from the Venn diagram analysis were further analyzed using Cytoscape version 3.10.2,^39^ an open-source platform for visualizing protein interaction networks. The STRING app version 2.2.0^40^ within Cytoscape with a score cutoff set to 0.4 was used to construct a protein-protein interaction network, based on known and predicted interactions. To identify the most central and potentially significant genes within this network, the CytoHubba plugin version 0.1 was used. The maximal clique centrality algorithm, known for its effectiveness in identifying hub genes,^41^ was applied to rank the genes within the network. The top 10 hub genes identified through this process were selected for further experimental validation, as their central role in the network suggests they may be critical regulators in the biological processes under study.

### Immune Cell Profiling and Deconvolution Using CIBERSORTx

CIBERSORTx version 1.0^42^ was used for immune cell deconvolution in human and mouse data sets. Gene expression data from selected GEO data sets were first normalized via quantile normalization to ensure cross-platform consistency.

### Empagliflozin Treatment Protocol *in Vivo*

Dystrophin-deficient *mdx* mice on the BL10 background (C57BL/10ScSn-Dmd^*mdx*^/J) and healthy wild-type (WT) control mice (C57BL/10ScSnJ) in an age range between 16 and 23 weeks were used. These two mouse lines were originally purchased from Charles River Laboratories (Wilmington, MA). For rats, dystrophin-deficient DMD^*mdx*^ Sprague-Dawley rats^43^ from INSERM–Center for Research in Transplantation and Translational Immunology UMR 1064 (Nantes, France) were used. As healthy controls, age-matched 9-month–old Sprague-Dawley rats were used. Only male animals were used, given the X-linked inheritance of DMD. Genotyping was performed using standard PCR assays.

Empagliflozin (MedChemExpress, Monmouth Junction, NJ; catalog number HY-15409) was dissolved in dimethyl sulfoxide to obtain a 50 mg/mL stock solution. Then, the stock solution was diluted in drinking water. For mice, the treatment started at 12 weeks of age and lasted for 4 weeks, with a final EMPA concentration of 0.1 mg/mL (0.2% dimethyl sulfoxide). In consideration of a mean water intake of 5 mL/day and a mean body weight of 33 g, the EMPA concentration of 0.1 mg/mL in the drinking water resulted in an EMPA dose of approximately 30 mg/kg body weight per day, a dose lying within the standard range for studies with mice.^22^ In rats, treatment was initiated at 5 months of age and continued for 4 months, with a dosage of 10 mg/kg body weight per day. Animals in the control groups were administered drinking water containing 0.2% dimethyl sulfoxide for either 4 weeks or 4 months, respectively.

### Experimental Validation of Hub Gene Expression via Real-Time Quantitative PCR

From the top 10 hub genes, the selection was refined to six by minimizing redundancy and prioritizing the most promising candidates identified through DE and WGCNA. Experimental validation was performed via quantitative RT-PCR on skeletal muscle tissue obtained from mouse and rat models of DMD treated with EMPA, alongside respective controls, providing critical empirical evidence to bridge the gap between *in silico* predictions and biological relevance.

### Quantitative Gene Expression Analysis in Skeletal Muscle Tissue

Total RNA was extracted from rat and mouse quadriceps muscle samples using the PureLink RNA Mini Kit (ThermoFisher, Waltham, MA) and quantified by Tecan reader using the NanoQuant plate (Tecan Trading AG, Männedorf, Switzerland). Total RNA was reversed transcribed using the QuantiTec-Reverse Transcription kit (Qiagen, Hilden, Germany) for a final concentration of 2 μg. Samples were analyzed in technical duplicates using a 20 μL reaction volume with a 1:5 dilution of cDNA in master mix solution. The initial denaturation step of 3 minutes at 95°C was followed by 40 cycles of 15 seconds at 95°C, 30 seconds at the annealing temperature, and 40 seconds at 72°C, using a CFX-Opus96 thermocycler with the accompanying CFX Manager software version 2.1 (Bio-Rad Laboratories Inc., Hercules, CA) for relative quantification using the cycle quantification (Cq) values. Specific rat primers (Microsynth GmbH, Vienna, Austria) and PerfeCTa SYBR Green FastMix (QuantaBio, Beverly, MA) were used according to the manufacturer's instructions to produce the master mix solution. The primer sequences are listed in Table 1.

**Table 1:** List of Primer Sequences Used for Gene Expression Analysis by Real-Time Quantitative PCR

Species	Gene name	Forward sequence	Reverse sequence	Annealing temperature, °C
Rat	*Hprt*	5′-CCCAGCGTCGTGATTAGTGATG-3′	5′-TTCAGTCCTGTCCATAATCAGTCC-3′	60
Rat	*Eef2*	5′-ATGAGGGCAAGATGAAGCTG-3′	5′-ATGAAGGACGGGATGGTTCAC-3′	60
Rat	*Col6A2*	5′-AGAACTTCTACAAGGCACGGC-3′	5′-GAGATGGTAGAGCATGGCGG-3′	62
Rat	*Pcolce*	5′-CCTCACTGTCACCGTCAGTC-3′	5′-TGCTTGCAGGGCACATAGAA-3′	62
Rat	*Sparc*	5′-AGGCATGGGCAGACCAATAC-3′	5′-CCCAGCAAGAGCCTGAAAGT-3′	62
Rat	*Timp1*	5′-ATAGTGCTGGCTGTGGGGTGTG-3′	5′-TGATCGCTCTGGTAGCCCTTCTC-3′	62
Rat	*Actb*	5′-CACTTTCTACAATGAGCTGCG-3′	5′-CTGGATGCCTACGTACATGG-3′	62
Rat	*Tgfb1*	5′-ACTCCCAACTACAGAAAAGCA-3′	5′-ATGACAGTGCGGTTATGGCA-3′	61
Rat	*Serpinh1*	5′-CCCAGCCCTCACAGGTCC-3′	5′-TGGCTTTACCACCCAGTGAC-3′	61
Mouse	*Eef2*	5′-CAGAAGTACCGTTGTGAGCTGC-3′	5′-GTCAGAGGTTGGCACCATCTTG-3′	60
Mouse	*Col6a2*	5′-TGGTCAACAGGCTAGGTGCCAT-3′	5′-TAGACAGGGAGTTGACTCGCTC-3′	62
Mouse	*Pcolce*	5′-GAGTGACGACTCAAAGAGGCT-3′	5′-AAGCCATCTGCGGTGACACTGA-3′	60
Mouse	*Sparc*	5′-CACCTGGACTACATCGGACCAT-3′	5′-CTGCTTCTCAGTGAGGAGGTTG-3′	60
Mouse	*Timp1*	5′-TATCCGGTACGCCTACACCC-3′	5′-TGGGCATATCCACAGAGGCT-3′	60
Mouse	*Actb*	5′-CATTGCTGACAGGATGCAGAAGG-3′	5′-TGCTGGAAGGTGGACAGTGAGG-3′	60
Mouse	*HSP47*	5′-TGAGGTCACCAAGGATGTGGAG-3′	5′-ATGAAGCCACGGTTGTCCACCA-3′	62
Mouse	*Tgfb1*	5′-GGCGAAGGCATTACAGTGTT-3′	5′-GGCCTGTCTCGAGGAATTAGG-3′	62
Mouse	*Lox1*	5′-CATACGTGCAGAGAGCCCAT-3′	5′-GCACTCGGAGGTCATAGTCG-3′	62
Mouse	*Glut1*	5′-GCTTCTCCAACTGGACCTCAAAC-3′	5′-ACGAGGAGCACCGTGAAGATGA-3′	60
Mouse	*Glut4*	5′-GGTGTGGTCAATACGGTCTTCAC-3′	5′-AGCAGAGCCACGGTCATCAAGA-3′	60
Mouse	*Cd36*	5′-GGACATTGAGATTCTTTTCCTCTG-3′	5′-GCAAAGGCATTGGCTGGAAGAAC-3′	60
Mouse	*Rps18*	5′-CGGAAAATAGCCTTCGCCATCAC-3′	5′-ATCACTCGCTCCACCTCATCCT-3′	60

### Functional Performance: Grip Strength Test

Grip strength was assessed using a grip strength meter (Ugo Basile, Gemonio, Italy). Three grip strength measurements for forelimbs were taken for 6 to 10 mice per group on a strength meter and averaged. The grip strength measurements were collected in the morning hours; force measurements (g), grip duration (seconds), and force normalized to body weight (seconds/g weight) data were recorded.

### Histologic Analysis, Immunohistochemistry, and Image Acquisition

Formalin-fixed, paraffin-embedded tissue sections from the mouse quadriceps muscle sample tissue were hematoxylin and eosin stained. The extent of fibrosis in skeletal muscle sections was visualized by Masson-Goldner staining (Masson-Goldner staining kit; Sigma-Aldrich/Merck, Darmstadt, Germany). Images were acquired by microscopy (Olympus VS120 Virtual Slide Microscope System; Olympus, Tokyo, Japan) and captured by digital camera (AVT PIKE F-505C VC 50; Allied Vision Technologies, Stadtroda, Germany). The extent of fibrosis was assessed by QuPath software version 0.5.1 (*https://qupath.github.io*). Immunohistochemistry streptavidin-biotin immunostaining for CD68 of paraffin-embedded tissue sections was performed with antibodies against CD68 (1:100; ED1; ab31630; Abcam, Cambridge, MA) to assess tissue macrophage density, as described previously.^44^ Primary antibodies were detected with biotinylated secondary antibodies (Vector Laboratories, Burlingame, CA) and peroxidase-conjugated streptavidin (Dako, Glostrup, Denmark), developed with 3,3′-diaminobenzidine (Vector Laboratories), counterstained with hematoxylin, dehydrated, and mounted in DPX (Merck, Darmstadt, Germany). Digitized images were generated with a slide scanner (VS120; Olympus, Hamburg, Germany) using Olympus-OlyVIA software version 4.2. In addition, nuclear localization (central versus peripheral) was assessed in skeletal muscle sections using hematoxylin and eosin staining. Three regions of equal size (300 × 300 μm) were randomly selected within each section, and the number of nuclei located in the periphery or center of myofibers was quantified. This measure is important in DMD, as the presence of centrally localized nuclei reflects ongoing cycles of muscle fiber degeneration and regeneration, serving as a histologic marker of disease severity and progression.^45^

### Quantification of Circulating TGF-β1

Quantification of circulating transforming growth factor (TGF)-β1 in mouse plasma was performed using a sandwich enzyme-linked immunosorbent assay (KE10005; Proteintech, Rosemont, IL). To activate latent TGF-β1, plasma samples were treated with half the sample volume of 1 N HCl and incubated for 10 minutes at room temperature, followed by neutralization with an equal volume of 1.2 N NaOH in 0.5 mol/L HEPES buffer. The neutralized samples were diluted 1:400 in the manufacturer's sample diluent and subjected to enzyme-linked immunosorbent assay, according to the supplier's protocol.

### Statistical Analysis

Real-time quantitative PCR and fibrosis data were analyzed using GraphPad Prism version 10.3.1 (GraphPad Software, Boston, MA). For comparisons between two groups, a two-tailed unpaired *t*-test was applied, and for comparisons among three groups, one-way analysis of variance followed by the Tukey *post hoc* test was used. The resulting CIBERSORTx outputs were then analyzed using GraphPad Prism version 10.3.1 and statistically compared using two-way analysis of variance. Data are expressed as means ± SEM, and significance was defined at *P* < 0.05 (∗*P* < 0.05, ∗∗*P* < 0.01, ∗∗∗*P* < 0.001, and ∗∗∗∗*P* < 0.0001).

### Animal Ethical Approval

The investigation conformed to the guiding principles of the Declaration of Helsinki and coincides with the rules of the Animal Welfare Committee of the Medical University of Vienna (Vienna, Austria). The experimental protocols were approved by the Austrian Science Ministry. The respective ethics vote has the following number: BMWFW-GZ 2020-0.570.648.

## Results

**Figure 1:**
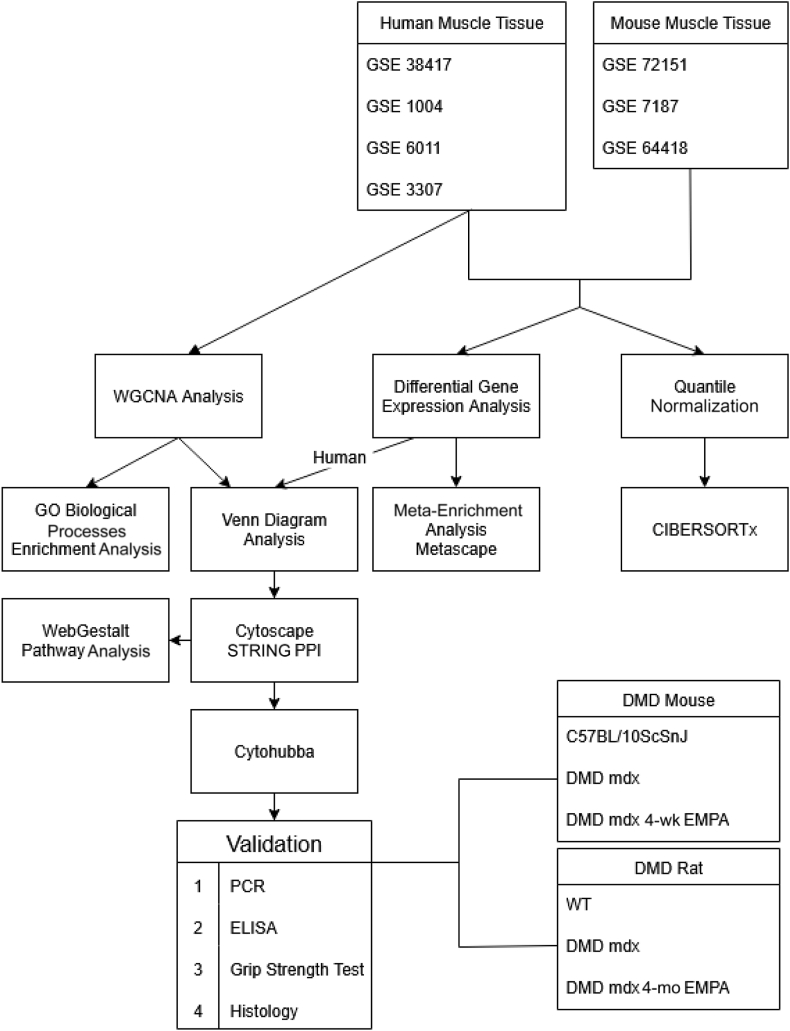
Overview of the study workflow. The flow diagram illustrates the comprehensive method used in this study. It begins with the collection of DMD microarray data sets from the Gene Expression Omnibus database, followed by differential expression analysis (DE) and weighted gene co-expression network analysis (WGCNA) across four human data sets. Venn diagram analysis was used to identify overlapping genes between DE and WGCNA results, leading to a set of 66 key genes. These genes were further analyzed through enrichment analysis using WebGestalt (Web-Based Gene Set Analysis Toolkit) and network analysis with Cytoscape and CytoHubba to identify central hub genes. The most promising targets were then validated experimentally using real-time quantitative PCR in animal tissue samples of mice and rats, supported by immune cell profiling with CIBERSORTx. The diagram provides a visual representation of how each method contributes to the identification and validation of potential gene targets. Links: *https://www.ncbi.nlm.nih.gov/geo/query/acc.cgi?acc=GSE38417*; accession number GSE38417; *https://www.ncbi.nlm.nih.gov/geo/query/acc.cgi?acc=GSE1004*; accession number GSE1004; *https://www.ncbi.nlm.nih.gov/geo/query/acc.cgi?acc=GSE6011*; accession number GSE6011; *https://www.ncbi.nlm.nih.gov/geo/query/acc.cgi?acc=GSE3307*; accession number GSE3307; *https://www.ncbi.nlm.nih.gov/geo/query/acc.cgi?acc=GSE72151*; accession number GSE72151; *https://www.ncbi.nlm.nih.gov/geo/query/acc.cgi?acc=GSE7187*; accession number GSE7187; and *https://www.ncbi.nlm.nih.gov/geo/query/acc.cgi?acc=GSE64418*; accession number GSE64418. ELISA, enzyme-linked immunosorbent assay; EMPA, empagliflozin; GO, Gene Ontology; PPI, protein-protein interaction; WT, wild type.

This study used a comprehensive *in silico* and experimental approach to identify and validate key gene targets associated with skeletal muscle fibrosis, and inflammation in DMD as well as to validate the anti-fibrotic efficacy of SGLT2i in the process. Publicly available microarray data on GEO were collected and analyzed for differential expression and co-expression across multiple human data sets. Overlapping genes, visualized through Venn diagram, were further examined using enrichment and network analyses to pinpoint potential hub genes. Six of the top 10 targets were then subjected to experimental validation via PCR in a preclinical model of DMD (Figure 1).

### Cross-Species Metascape Enrichment Analysis Reveals Key Pathways in Tissue Remodeling and Immune Responses

**Figure 2:**
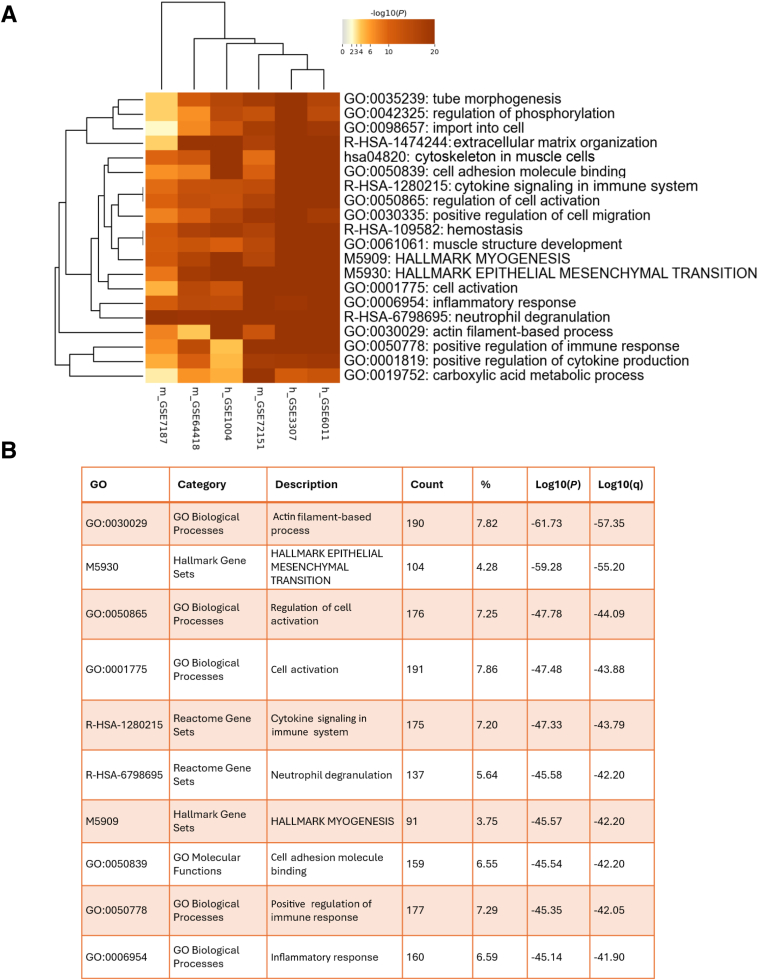
Metascape enrichment analysis of differentially expressed genes across human and mouse data sets. **A:** The heat map displays the hierarchical clustering of the top enriched Gene Ontology (GO) terms and pathways identified across the seven studies (four human and three mouse data sets). Clustering of common biological processes, such as cytokine signaling and neutrophil degranulation, shared across species. **B:** Top enriched terms and pathways, sorted by false discovery rate. The analysis reveals significant enrichment of processes related to extracellular matrix organization, immune responses, and cytoskeletal organization, which are important in the context of the condition being studied. Links: Metascape, *https://metascape.org/gp/index.html#/main/step1*; Molecular Signatures Database (MSigDB), *https://www.gsea-msigdb.org/gsea/msigdb/index.jsp*; *https://www.ncbi.nlm.nih.gov/geo/query/acc.cgi?acc=GSE6011*; accession number GSE6011; *https://www.ncbi.nlm.nih.gov/geo/query/acc.cgi?acc=GSE3307*; accession number GSE3307; *https://www.ncbi.nlm.nih.gov/geo/query/acc.cgi?acc=GSE72151*; accession number GSE72151; *https://www.ncbi.nlm.nih.gov/geo/query/acc.cgi?acc=GSE1004*; accession number GSE1004; *https://www.ncbi.nlm.nih.gov/geo/query/acc.cgi?acc=GSE64418*; accession number GSE64418; and *https://www.ncbi.nlm.nih.gov/geo/query/acc.cgi?acc=GSE7187*; accession number GSE7187.

Metascape enrichment analysis was performed to explore the biological relevance of the DEGs identified across human and mouse data sets. The analysis revealed consistent enrichment of pathways related to extracellular matrix organization, cytokine signaling, and immune system responses across both species (Figure 2A).

Among the top enriched categories, pathways such as cytokine signaling in the immune system, neutrophil degranulation, and actin filament-based processes were consistently observed across data sets (Figure 2B). These findings emphasize the involvement of both structural and immune-related processes, offering a comprehensive overview of the molecular mechanisms potentially contributing to the observed muscle dysfunction in DMD. The detailed enrichment data, presented in the table (Figure 2B), underlined the importance of these pathways and provides a base for subsequent in-depth investigations.

### Weighted Gene Co-Expression Network Analysis Reveals Key Gene Modules and Biological Processes in DMD

**Figure 3:**
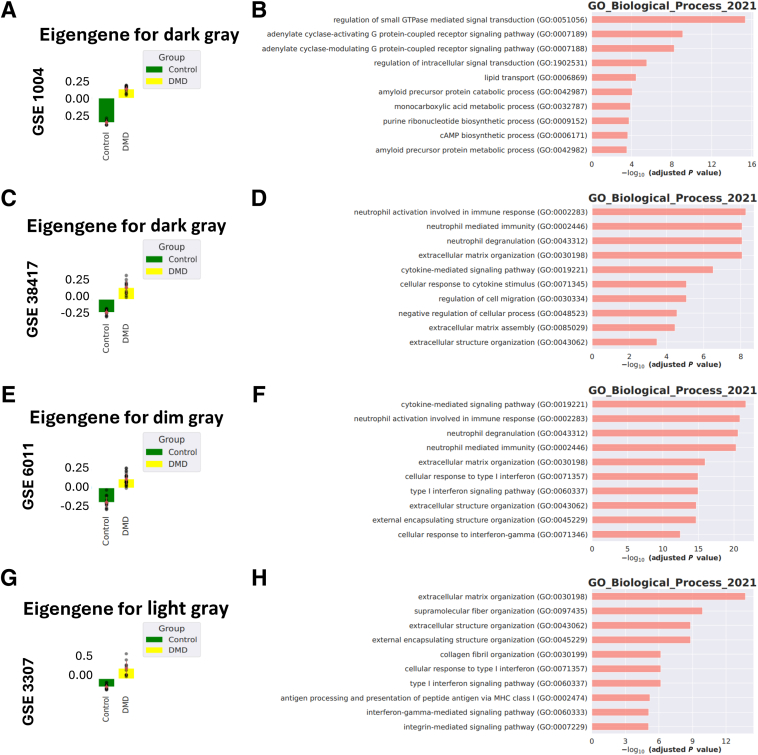
Weighted gene co-expression network analysis of gene modules across human data sets. **A**, **C**, **E**, and **G:** Eigengene expression plots for the most positively correlated modules identified in each data set, showing clear distinctions between control and DMD groups. **B**, **D**, **F**, and **H:** Enrichment analysis results for the positively correlated modules, focusing on Gene Ontology (GO) Biological Processes. The analysis reveals significant involvement of processes related to extracellular matrix organization, immune response, and signaling pathways. Links: *https://www.ncbi.nlm.nih.gov/geo/query/acc.cgi?acc=GSE38417*; accession number GSE38417; *https://www.ncbi.nlm.nih.gov/geo/query/acc.cgi?acc=GSE1004*; accession number GSE1004; *https://www.ncbi.nlm.nih.gov/geo/query/acc.cgi?acc=GSE6011*; accession number GSE6011; and *https://www.ncbi.nlm.nih.gov/geo/query/acc.cgi?acc=GSE3307*; accession number GSE3307. MHC, major histocompatibility complex.

WGCNA was conducted across four human data sets to identify gene modules associated with the condition under study. The analysis focused on the most positively correlated modules, revealing clear distinctions between the control and DMD groups across all data sets (Figure 3, A, C, E, and G). These modules were selected on the basis of their strong correlation with Duchenne muscular dystrophy.

Enrichment analysis of the identified modules highlighted several biological processes that are consistently associated across the data sets (Figure 3, B, D, F, and H). Similar to the findings of the Metascape enrichment analysis, WGCNA analysis confirmed that the significance of the processes, including extracellular matrix organization, immune responses, and signaling pathways, was key in tissue remodeling and inflammation in skeletal muscle dysfunction in DMD. These findings provided a foundation for further exploration of the molecular mechanisms underlying the observed phenotypes.

### Integrating Multi–Data Set Gene Discovery: Venn Diagram Analysis and Pathway Enrichment

**Figure 4:**
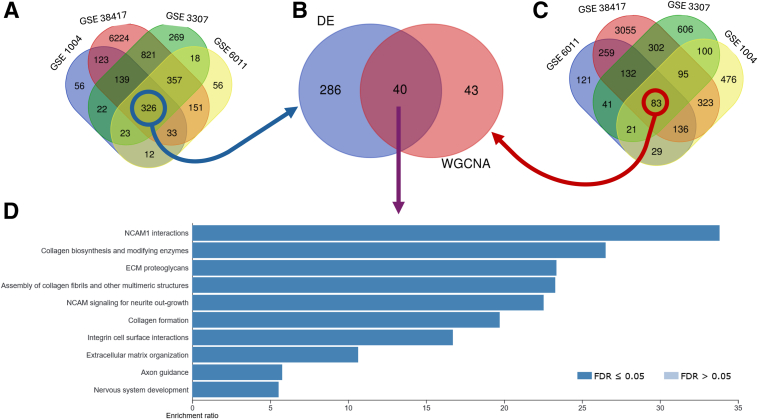
Venn diagram and enrichment analysis of overlapping genes. **A:** Venn diagram showing the overlap of differentially expressed genes across the four human data sets, resulting in 326 overlapping genes. **B:** Venn diagram combining the results from differential expression (DE) analysis and weighted gene co-expression network analysis (WGCNA), identifying 40 genes that overlap between both analyses. **C:** Venn diagram showing the overlap of 83 WGCNA-identified genes, including those present in at least three of the four modules. **D:** Bar plot generated by WebGestalt (Web-Based Gene Set Analysis Toolkit), illustrating the top enriched pathways and processes for the 40 overlapping genes, with significant categories including extracellular matrix organization, integrin signaling, and nervous system development. Links: *https://www.ncbi.nlm.nih.gov/geo/query/acc.cgi?acc=GSE1004*; accession number GSE1004; *https://www.ncbi.nlm.nih.gov/geo/query/acc.cgi?acc=GSE38417*; accession number GSE38417; *https://www.ncbi.nlm.nih.gov/geo/query/acc.cgi?acc=GSE3307*; accession number GSE3307; and *https://www.ncbi.nlm.nih.gov/geo/query/acc.cgi?acc=GSE6011*; accession number GSE6011. ECM, extracellular matrix; FDR, false discovery rate; NCAM, neural cell adhesion molecule.

To identify genes consistently associated across multiple data sets and analytical approaches, a Venn diagram analysis was conducted. The first Venn diagram (Figure 4A) highlighted the overlap of DEGs across all four human data sets, resulting in a set of 326 genes. The second diagram (Figure 4C) focused on genes identified through WGCNA, yielding a total of 83 genes. The final Venn diagram (Figure 4B) combined the results from both DE and WGCNA analyses, revealing a set of 40 overlapping genes that are consistently implicated across the different methods and data sets.

Subsequent enrichment analysis of these 40 genes was conducted using WebGestalt, focusing on key biological processes and pathways (Figure 4D). Similarly, the analysis identified significant enrichment in pathways related to extracellular matrix organization, integrin signaling, nervous system development, and other key processes. These pathways played a crucial role in tissue remodeling, cellular interactions, and homeostasis.

### Protein-Protein Interaction Network and Identification of Key Hub Genes

**Figure 5:**
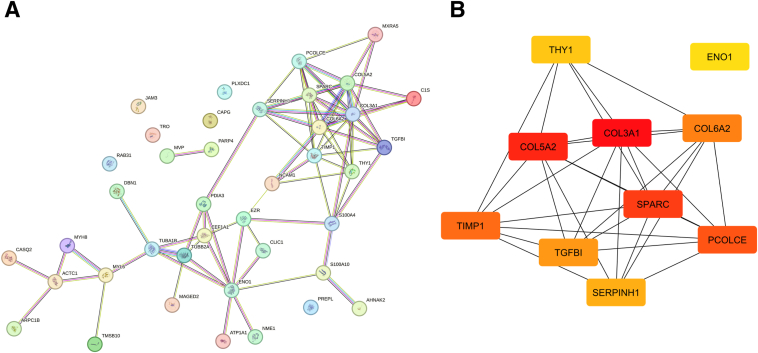
Network visualization and hub gene analysis of overlapping genes. **A:** Protein-protein interaction network constructed using the STRING database for the 66 overlapping genes identified in the Venn diagram analysis. The network highlights the interactions among these genes, providing insights into their potential roles in cellular processes. **B:** Visualization of the top 10 hub genes identified by the maximal clique centrality algorithm in CytoHubba, showcasing their central roles in the network. The hub genes include *COL3A1*, *COL5A2*, *COL6A2*, *PCOLCE*, *SERPINH1*, *SPARC*, *TGFBI*, *THY1*, *ENO1*, and *TIMP1*.

To investigate the protein-protein interactions among the 40 overlapping genes identified through Venn diagram analysis, the Search Tool for the Retrieval of Interacting Genes/Proteins (STRING) database (*https://string-db.org*, last accessed August 12, 2024) was used to construct a protein-protein interaction network (Figure 5A). This network visualization offered a comprehensive overview of the connectivity and interaction landscape of these genes, emphasizing their potential involvement in muscle fibrosis and inflammation in DMD. Further analysis with CytoHubba using the maximal clique centrality algorithm identified the top 10 hub genes within the network, which are displayed in Figure 5B. These hub genes were *THY1*, *COL3A1*, *ENO1*, *COL5A2*, *COL6A2*, *PCOLCE*, *SERPINH1*, *SPARC*, *TGFBI*, and *TIMP1*. To experimental validation, six hub genes were selected for further analysis, excluding *THY1* and *ENO1* because of their broader, less disease-specific roles. *COL3A1* and *COL5A2* were also excluded to prevent redundancy in collagen-related gene analysis. This selection enabled a focus on genes more closely linked to ECM remodeling and fibrosis based on the main hypothesis of the study.

### Comparative Immune Profiling in Duchenne Muscular Dystrophy: Insights from CIBERSORTx Analysis

**Figure 6:**
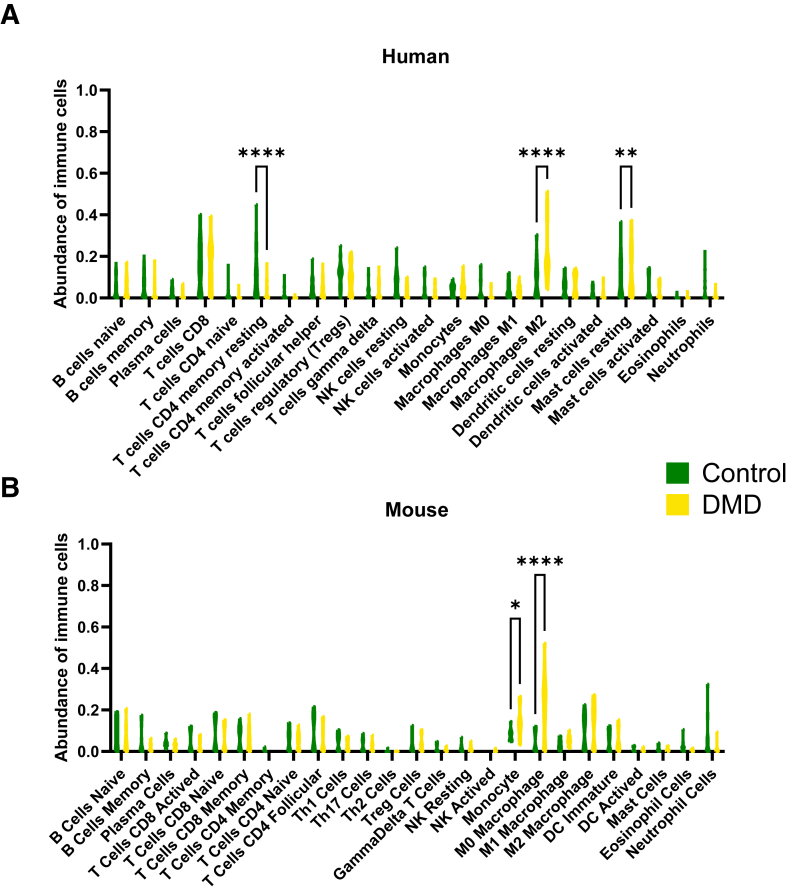
Immune cell profiling in human and mouse data sets using CIBERSORTx. **A:** Immune cell composition in human DMD samples compared with healthy controls, showing a significant decrease in resting CD4^+^ T cells and resting mast cells, and a significant increase in M2 macrophages. **B:** Immune cell composition in the DMD mouse model compared with healthy controls, showing a significant increase in monocytes and macrophages. The results highlight both shared and species-specific immune responses in DMD, with macrophage populations playing a key role across both data sets. Differences in the severity of the DMD phenotype between humans and mice, along with age-related differences, may influence these observed immune alterations. Data are given as means ± SEM (**A** and **B**). *n* = 44 human control (**A**); *n* = 61 human DMD (**A**); *n* = 14 mouse control (**B**); *n* = 15 mouse DMD (**B**). ∗*P* < 0.05, ∗∗*P* < 0.01, and ∗∗∗∗*P* < 0.0001. DC, dendritic cell; NK, natural killer; Th1, type 1 helper T cell; Th17, type 17 helper T cell.

The analysis was performed to profile the immune cell populations in both human and mouse data sets, comparing healthy controls with DMD samples (Figure 6). This analysis aimed to elucidate the immune landscape associated with DMD and identify potential similarities and differences between the two species.

In the human muscle data sets (Figure 6A), significant immune alterations were observed in patients with DMD compared with healthy controls. Notably, there was a significant decrease in resting CD4^+^ T cells and resting mast cells, along with a marked increase in M2 macrophages. These findings suggested a shift toward a more inflammatory state, commonly associated with tissue remodeling and fibrosis. In the DMD mouse model (Figure 6B), similar immune changes were observed, albeit with notable differences. Specifically, DMD mice exhibited a significant increase in both monocytes and macrophages compared with healthy controls.

### EMPA Treatment Modulates the Expression of Key Hub Genes in Small Animal Models of DMD

The gene expression of selected hub genes, *Col6a2*, *Sparc*, *Serpinh1*, *Timp1*, *Pcolce*, and *Tgfb1*, was assessed by real-time quantitative PCR in skeletal muscle tissue from C57BL/10ScSnJ, vehicle-treated DMD, and EMPA-treated DMD (EMPA + DMD) mice.

**Figure 7:**
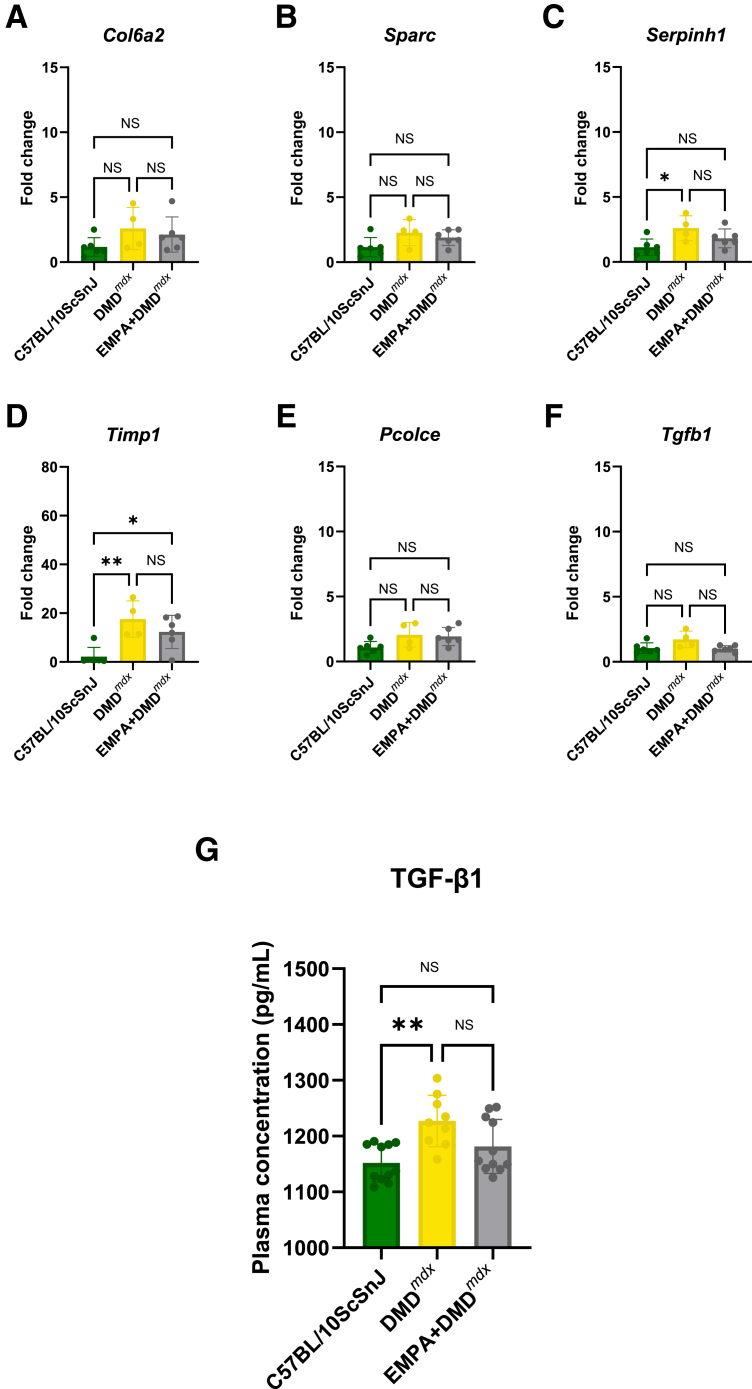
Expression of key hub genes in mouse model of DMD after the empagliflozin (EMPA) treatment. **A**–**F:** Real-time quantitative PCR analysis of genes involved in extracellular matrix remodeling and fibrosis in C57BL/10ScSn (healthy control), DMD, and EMPA-treated DMD (EMPA + DMD) mouse models. **A:***Col6a2* expression shows no significant changes across groups. **B:***Sparc* expression trends toward an increase in DMD compared with controls (*P* = 0.0975), but no significant effect of EMPA treatment is observed (*P* = 0.7305). **C:***Serpinh1* expression is significantly elevated in DMD compared with healthy controls (*P* = 0.0245), with no significant reduction following EMPA treatment (*P* = 0.2797). **D:***Timp1* expression is significantly increased in DMD compared with controls (*P* = 0.0043). **E:***Pcolce* expression does not show significant differences across groups. **F:***Tgfb1* expression trends toward a reduction in EMPA-treated mice compared with DMD (*P* = 0.0516), although the change does not reach statistical significance. **G:** Plasma concentrations of transforming growth factor (TGF)-β1, measured by enzyme-linked immunosorbent assay, are significantly elevated in DMD mice compared with controls (*P* = 0.0015), and show a nonsignificant reduction following EMPA treatment (*P* = 0.061). Data are given as means ± SEM (**A**–**G**). *n* = 6 C57BL/10ScSnJ and EMPA + DMD^*mdx*^ (**A**–**G**); *n* = 4 DMD^*mdx*^ (**A**–**G**). ∗*P* < 0.05, ∗∗*P* < 0.01. NS, nonsignificant.

Among the genes examined, *Timp1* expression was significantly elevated in DMD mice compared with WT controls (*P* = 0.0043) and was significantly reduced following EMPA treatment (*P* = 0.0300) (Figure 7D). *Serpinh1* (*P* = 0.0245) was significantly up-regulated in DMD mice relative to WT, with a tendency toward increased expression following EMPA treatment (*P* = 0.2797) (Figure 7C). *Tgfb1* showed a trend toward increased expression in DMD (*P* = 0.0730), which was mitigated after EMPA treatment (*P* = 0.0516) (Figure 7F). In contrast, *Col6a2*, *Sparc*, and *Pcolce* did not show statistically significant differences between the groups (Figure 7, A, B, and E). To determine whether changes in gene expression were reflected at the systemic level, plasma concentrations of TGF-β1 were measured across the experimental groups. Consistent with the transcriptional trend observed in muscle tissue, TGF-β1 levels were significantly elevated in DMD mice compared with WT controls (*P* = 0,0015). EMPA treatment reduced circulating TGF-β1 levels; however, this reduction did not reach statistical significance (*P* = 0.0617) (Figure 7G).

**Figure 8:**
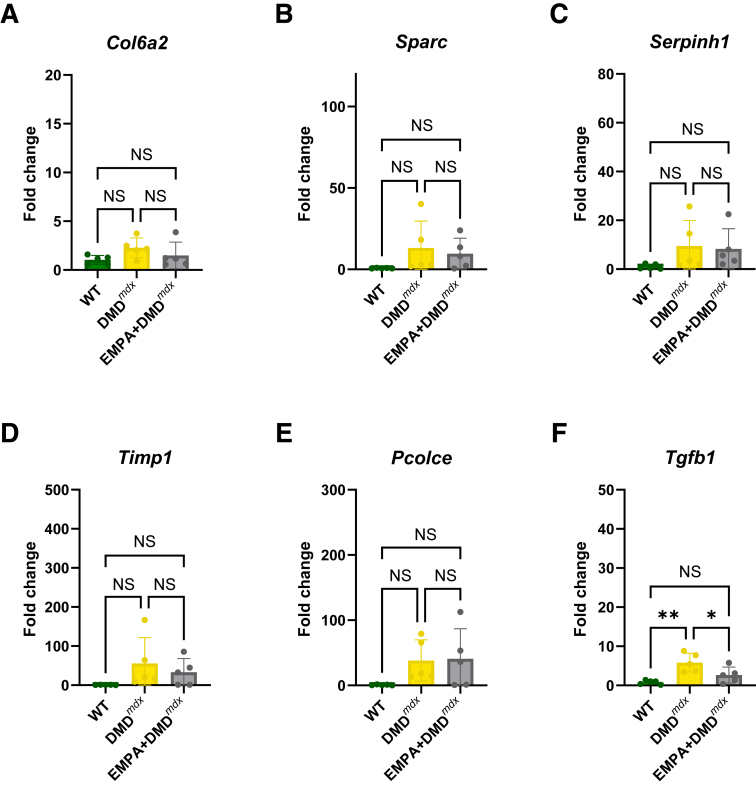
Expression of key hub genes in DMD rat models with and without empagliflozin (EMPA) treatment. **A**–**F:** Real-time quantitative PCR analysis of key hub genes in wild-type (WT), DMD, and EMPA-treated DMD (EMPA + DMD) rat models. **A:***Col6a2* expression shows no significant changes across groups (*P* = 0.1769 for WT versus DMD, and *P* = 0.4715 for DMD versus EMPA + DMD). **B:***Sparc* expression exhibits a trend toward increase in DMD compared with WT (*P* = 0.2138) but shows no significant differences with EMPA treatment (*P* = 0.8716). **C:***Serpinh1* expression does not show significant changes across groups (*P* = 0.2345 for WT versus DMD, and *P* = 0.9693 for DMD versus EMPA + DMD). **D:***Timp1* expression trends toward an increase in DMD compared with WT (*P* = 0.1531) but shows no statistically significant reduction with EMPA treatment (*P* = 0.6956). **E:***Pcolce* expression shows no significant differences across groups (*P* = 0.1650 for WT versus DMD, and *P* = 0.99 for DMD versus EMPA + DMD). **F:***Tgfb1* expression is significantly increased in DMD compared with WT (*P* = 0.0026) and is significantly reduced following EMPA treatment (*P* = 0.0454 for DMD versus EMPA + DMD), suggesting modulation of fibrotic signaling. Data are given as means ± SEM (**A**–**F**). *n* = 5 per group (**A**–**F**). ∗*P* < 0.05, ∗∗*P* < 0.01. NS, nonsignificant.

To extend this *in silico* analysis of EMPA as a novel therapeutic for skeletal muscle dysfunction in DMD, the expression of key hub genes *Col6a2*, *Sparc*, *Serpinh1*, *Timp1*, *Pcolce*, and *Tgfb1* in WT, untreated DMD, and EMPA-treated DMD (EMPA + DMD) rats was quantified using real-time quantitative PCR. Among the genes assessed, *Col6a2* and *Sparc*, which showed no significant changes in the mouse model (Figure 7, A and B), also remained unchanged in rats (*P* = 0.7837 and *P* = 0.4274 for WT versus DMD, respectively) (Figure 8, A and B). Similarly, *Pcolce* did not exhibit significant differential expression in rats (*P* = 0.1850 for WT versus DMD, *P* = 0.2035 for DMD versus EMPA + DMD) (Figure 8E), consistent with the mouse model (Figure 7E). This aligned with the near-significant reduction observed in the mouse model (*P* = 0.0516). Although *Serpinh1* was significantly up-regulated in DMD mice compared with WT animals, no significant change in *Serpinh1* expression was observed in the rat model (Figure 8C). *Timp1* expression trends toward an increase in DMD compared with WT (*P* = 0.1531) but shows no statistically significant reduction with EMPA treatment (*P* = 0.6956) (Figure 8D). Importantly, *Tgfb1* was the only gene that consistently responded to EMPA treatment across both species (Figure 8F).

### Grip Strength Testing Reveals Impaired Performance in DMD^*mdx*^ Mice

**Figure 9:**
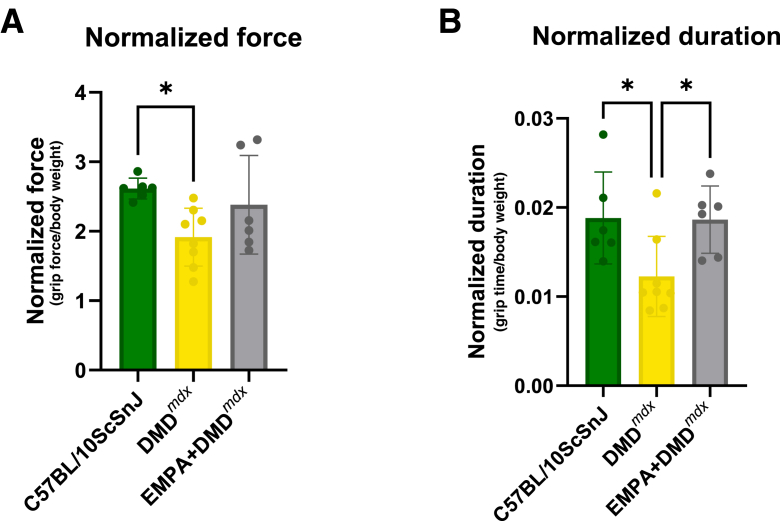
Empagliflozin (EMPA) improves grip endurance in DMD^*mdx*^ mice. **A:** Normalized peak grip force (peak force/body weight). DMD^*mdx*^ mice display lower normalized force than wild type; EMPA-treated DMD^*mdx*^ do not differ significantly from either group. **B:** Normalized grip duration (grip time/body weight). DMD^*mdx*^ mice show shorter normalized duration than wild type, whereas EMPA significantly increases duration versus untreated DMD^*mdx*^, reaching values comparable to wild type. Data are given as means ± SEM (**A** and **B**). *n* = 6 C57BL/10ScSnJ and DMD^*mdx*^ EMPA-treated mice (**A** and **B**); *n* = 8 DMD^*mdx*^ (**A** and **B**). ∗*P* < 0.05.

Normalized peak force (peak force/body weight) was significantly reduced in DMD^*mdx*^ compared with wild-type C57BL/10ScSnJ mice (Figure 9A) (*P* < 0.05). EMPA-treated DMD^*mdx*^ animals showed intermediate values that were not significantly different from either wild-type or untreated DMD^*mdx*^. Normalized grip duration (grip time/body weight) was also reduced in DMD^*mdx*^ versus wild type (Figure 9B) (*P* < 0.05). Importantly, EMPA treatment significantly increased normalized duration relative to untreated DMD^*mdx*^ (Figure 9B) (*P* < 0.05), suggesting that EMPA improves muscle endurance capacity in DMD^*mdx*^ mice, partially rescuing functional deficits in grip maintenance despite limited effects on peak force generation. EMPA treatment not only improved normalized grip duration but also reduced central nucleation, suggesting a coordinated functional and structural benefit (Supplemental Figure S1). The rescue of nuclear localization toward the periphery indicated enhanced myofiber maturation and stability, which may underline the observed improvement in muscle endurance.

### Skeletal Muscle Fibrosis, Inflammation, and Metabolism

**Figure 10:**
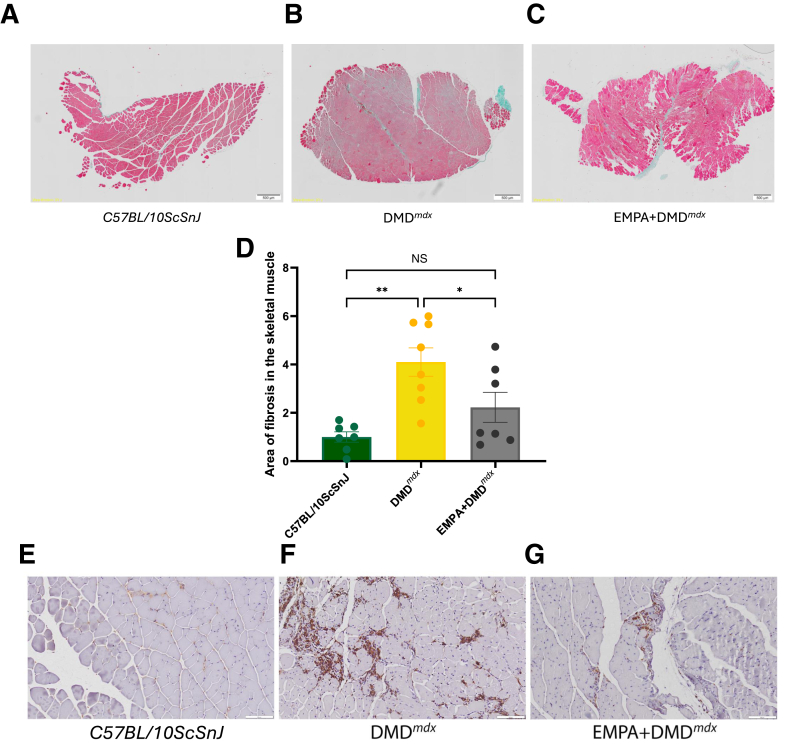
**A**–**C** and **E**–**G:** Fibrosis assessment in the skeletal muscle of wild-type group (**left panels**), DMD^*mdx*^ (**middle panels**), and DMD^*mdx*^ empagliflozin (EMPA)–treated mice (**right panels**). **A**–**C:** Representative photomicrographs of Masson-Goldner staining that highlights the collagen fibers in green, muscle cytoplasm in pink, and the nuclei in dark blue. **D:** Area of fibrosis in the skeletal muscle depicted as bar plot. **E**–**G:** Immunohistochemistry was performed to evaluate the influx of CD68^+^ macrophages in cardiac muscle. Representative photomicrographs of wild-type, DMD^*mdx*^, and EMPA + DMD^*mdx*^ mice are shown. Data are given as means ± SEM (**D**). *n* = 7 C57BL/10ScSnJ (**D**); *n* = 5 DMD^*mdx*^ (**D**); *n* = 4 DMD^*mdx*^ EMPA-treated mice (**D**). ∗*P* < 0.05, ∗∗*P* < 0.01. Scale bars: 500 μm (**A**–**C**); 100 μm (**E**–**G**). NS, nonsignificant.

In DMD^*mdx*^ mice, which develop pronounced muscle fibrosis as a hallmark of the disease, EMPA treatment led to a marked reduction in fibrotic deposition. Fibrosis was markedly elevated in vehicle-treated mdx mice compared with wild-type controls, reflecting the pathologic remodeling associated with dystrophic muscle. Notably, EMPA-treated mdx samples exhibited a significant decrease in fibrosis. In addition, no CD68^+^ cells were detected in the wild-type group (Figure 10). In contrast, numerous accumulations of CD68^+^ macrophages were present in the myocardial tissue of the mdx group. Importantly, treatment with EMPA was associated with reduced influx of CD68^+^ macrophages (Figure 10). These results suggested that EMPA can effectively attenuate disease-associated fibrotic remodeling and macrophage influx in dystrophic muscle, highlighting its potential as a therapeutic intervention. Recent studies highlighted SGLT2i, enhancing myocardial free fatty acid utilization while reducing free fatty acid uptake to mitigate lipotoxicity. Here, it was also addressed and investigated whether SGLT2i affected *Glut1*, *Glut4*, *Cd36,* and *Olr1* (Supplemental Figure S2). Interestingly, *Glut1* mRNA expression levels were markedly increased in mdx tissue, and this effect was reversed by EMPA treatment. However, no differences in *Glut4* or *Cd36* expression were observed between the groups (Supplemental Figure S2).

Collectively, the data indicated significant differences in collagen deposition among the groups, with DMD^*mdx*^ samples exhibiting elevated fibrosis compared with wild type and a notable reduction in EMPA-treated DMD^*mdx*^. These findings provided an objective record of the histologic alterations observed before further mechanistic discussion.

## Discussion

*In silico* approaches have become essential tools in modern drug discovery, enabling the rapid identification of molecular targets and candidate therapies by integrating computational models with large-scale omics data^46^ These strategies are particularly valuable for drug repurposing, offering a cost-effective alternative to traditional *in vivo* studies.^47^ In diseases like Duchenne muscular dystrophy, where drug development is hindered by complexity and rarity, such approaches can accelerate therapeutic discovery.^48^ For example, SGLT2 inhibitors—originally developed for diabetes—were predicted via computational models to modulate fibrotic pathways in DMD.^49^^,^^50^ This study is the first, to current knowledge, to explore the therapeutic potential of SGLT2i in this context. Using transcriptomic analysis, dysregulated pathways were identified in DMD skeletal muscle that may be modifiable by SGLT2i. These included ECM organization, cytokine signaling, and immune responses, consistent with prior research.^51^ From 40 overlapping genes, 10 hub genes emerged as key regulators of fibrosis and tissue remodeling. Six of these were selected for experimental validation, excluding those with broad or redundant roles (eg, *THY1*, *ENO1*, *COL3A1*, and *COL5A2*) to focus on ECM-specific mechanisms. Quantitative RT-PCR in mdx and DMD^*mdx*^ animal models confirmed dysregulation of several of these genes.^52^ EMPA treatment led to decreased expression of *Tgfb1* in rats and modulation of fibrotic and inflammatory markers, although *Timp1* remained elevated.^53^

Beyond hemodynamics, SGLT2 inhibitors can reprogram gene expression via miRNA networks. In hypertensive patients with type 2 diabetes, dapagliflozin shifted circulating miRNAs linked to vascular/renal biology—forming an epigenetic signature (eg, up-regulated miR-30e-5p, down-regulated miR-199a-3p).^54^ In frail heart failure with preserved ejection fraction + diabetes, empagliflozin improved an endothelial dysfunction miRNA profile (including reductions in profibrotic miR-21/miR-92 signatures), providing direct clinical evidence that SGLT2i modulate epigenetically active regulators relevant to fibrosis and inflammation.^55^ A recent review further frames how SGLT2i intersect with cardiac epigenetics (DNA/histone marks and noncoding RNAs) in arrhythmia/fibrosis biology.^56^ In DMD, several of these miRNAs converge on TGF-β/Smad and ECM programs; thus, the observed reductions in *Tgfb1* and fibrosis with EMPA are mechanistically consistent with miRNA-mediated post-transcriptional control.

Dystrophic skeletal muscle shows a shift toward glycolytic metabolism with impaired mitochondrial oxidative phosphorylation, especially in fast/glycolytic fibers, whereas promoting a slow/oxidative program [eg, via Peroxisome proliferator-activated receptor gamma coactivator 1-alpha (PGC-1α)] is protective.^57^ Notably, glucose uptake in skeletal muscle is predominantly glucose transporter type 4 (GLUT4) mediated, so a direct blockade of myocellular glucose influx by SGLT2i is unlikely; SGLT1/2 expression in muscle appears low/heterogeneous and context dependent.^58^ In the present study, skeletal muscle exhibits increased *Glut1* expression with unchanged *Glut4*, reflecting a shift toward basal, insulin-independent glucose uptake. This likely represents a compensatory mechanism to sustain energy supply in regenerating fibers under chronic injury and impaired insulin signaling. Reversal of *Glut1* up-regulation by EMPA suggests normalization of muscle metabolism. In humans, SGLT2i reprograms skeletal muscle fuel use toward greater fatty acid and ketone oxidation with reduced glycolytic flux, and positron emission tomography confirms increased muscle fatty acid uptake; in mice, empagliflozin activates an AMP-activated protein kinase–nicotinamide phosphoribosyltransferase (NAMPT)–PGC-1α low-nutrient transcriptional program in muscle.^59^^,^^60^ In dystrophic versus healthy muscle, such a shift could be double edged: ketones can support endurance by sparing glycolysis, yet mitochondrial deficits in DMD may constrain fatty acid/ketone oxidation—yielding net effects that plausibly diverge by genotype.^57^^,^^61^ Finally, fiber type–specific responses to SGLT2i have been observed (differences in slow versus fast muscles), aligning with endurance gain despite unchanged peak force and pointing to a metabolism-fibrosis-endurance axis worth direct testing in DMD.^62^

Although *COL6A2* and *SPARC* were overexpressed in human DMD data sets, they did not show consistent regulation in mdx mice, underlining species-specific differences.^63^^,^^64^ Despite these discrepancies, histologic assessment demonstrated that EMPA treatment significantly reduced fibrosis in DMD^*mdx*^ muscle, supporting its role in attenuating ECM remodeling.^22^^,^^53^ These findings align with previous reports of SGLT2i-mediated modulation of the AMP-activated protein kinase α/matrix metalloproteinase 9/TGF-β1/Smad pathway in other fibrotic contexts.^53^ Interestingly, *Serpinh1*—a key regulator of collagen folding—remained elevated after treatment, suggesting that EMPA primarily affects upstream fibrotic signaling rather than collagen maturation processes.^65^

To determine whether these local changes were reflected systemically, plasma TGF-β1 concentrations were measured. Consistent with transcriptional trends, DMD^*mdx*^ mice exhibited significantly elevated circulating TGF-β1 compared with wild-type controls. Although EMPA-treated animals showed a reduction in plasma TGF-β1, this did not reach statistical significance, possibly because of variability in systemic response or insufficient treatment duration.^53^ These findings indicate that local tissue-level changes may precede systemic shifts or require prolonged exposure for measurable impact.

Functional assessments revealed reduced grip strength and endurance in DMD^*mdx*^ mice, reflecting dystrophic muscle dysfunction.^66^ EMPA treatment did not significantly improve normalized peak force but did restore grip duration to wild-type levels, suggesting enhanced muscle endurance. The functional deficits observed in DMD^*mdx*^ mice, including reduced normalized grip force and duration, were accompanied by increased central nucleation, reflecting cycles of degeneration and regeneration typical of dystrophic muscle.^67^ EMPA treatment not only improved normalized grip duration but also reduced the proportion of centrally located nuclei, indicating a shift toward more mature and structurally stable myofibers; consequently, the restoration of peripheral nuclear positioning is widely recognized as a marker of enhanced muscle fiber integrity and regenerative efficiency. Thus, the parallel improvements in grip endurance and nuclear localization suggest that EMPA exerts dual benefits on both the structural and functional properties of dystrophic muscle. These findings support the notion that targeting cellular mechanisms contributing to myofiber stability may translate into measurable functional improvements in DMD.

In addition, the observed functional improvement may be partially explained by a reduction in inflammation and fibrosis and changes the metabolism. Histologic analysis further validated these findings, showing a marked collagen accumulation in DMD^*mdx*^ muscle, which was significantly reduced in EMPA-treated animals. This decrease in fibrotic burden aligns with molecular and functional data, reinforcing the anti-fibrotic potential of EMPA in DMD.^53^ Beyond effects on muscle and fibro-inflammation, there is a growing body of evidence that SGLT2i influence macrophage polarization and metabolism.^68^ For example, after myocardial infarction, dapagliflozin reduced fibrosis by promoting M2 via STAT3 signaling.^69^ In the present model, CD68 immunostaining revealed no detectable macrophages in wild-type myocardium, whereas a robust CD68^+^ accumulation in DMD^*mdx*^ hearts and a reduction with EMPA treatment (Figure 10) were observed. These findings support a role for SGLT2i that may limit the macrophage influx in dystrophic myocardium, consistent with prior reports of SGLT2i-mediated modulation of macrophage phenotype. In obesity, empagliflozin increased M2 macrophages in adipose tissue and liver while improving energetics,^70^ and ipragliflozin attenuated the M1/M2 ratio in adipose tissue.^71^ Mechanistically, SGLT2i can restrain glycolysis and shift macrophage programs (eg, down-regulating 6-phosphofructo-2-kinase/fructose-2,6-bisphosphatase 3 (PFKFB3) with reduced glycolytic flux and M2 skewing in nonalcoholic fatty liver disease models^68^ and down-regulating sodium-hydrogen exchanger 1 (NHE1) to prevent lipopolysaccharide-induced M1 polarization in inflammatory bowel disease).^72^ At the cellular bioenergetic level, empagliflozin activates AMP-activated protein kinase and improves macrophage energy metabolism.^73^ These shifts align with canonical immunometabolism in which M1 relies more on glycolysis, whereas M2 favors oxidative phosphorylation/fatty acid oxidation—with important context-dependent nuances.^74^, ^75^, ^76^ Notably, tissue context matters: in a renal injury model, empagliflozin reduced profibrotic CD206^+^ alternative macrophages and fibrosis,^77^ underlining that M2 subsets can be reparative or fibrogenic. Collectively, suppression of glycolytic flux (via PFKFB3/NHE1) with consequent AMP-activated protein kinase activation promotes M2 polarization, providing an immunometabolic mechanism that links the findings to TGF-β–driven extracellular matrix remodeling observed in DMD. Other interesting findings were the mRNA expression changes of Glut1 in dystrophic muscle. This may suggest a shift toward basal, insulin-independent glucose uptake, and treatment with EMPA reversed the Glut1 mRNA up-regulation, indicating improved metabolic efficiency, and reduce the muscle's reliance on Glut1, restoring a more physiological Glut4-dominant glucose uptake profile. These findings highlight the potential of metabolic modulation to alleviate energetic stress in dystrophic muscle. A cross-study comparative analysis underlines substantial context dependence in transcriptional regulation: cd36 demonstrates data set–specific variability, PFKFB3 is consistently down-regulated only within a subset of human cohorts, and oxidized low-density lipoprotein receptor 1 (OLR1) undergoes minor changes, whereas most mouse-derived effects remain nonsignificant (Supplemental Figure S3). These patterns suggest that regulatory dynamics of metabolic and inflammatory mediators are strongly influenced by experimental context and species background, highlighting the limitations of direct cross-species extrapolation.

Chronic inflammation arising from dystrophin deficiency drives persistent immune cell infiltration, which amplifies myofiber damage and accelerates the progression of fibrotic remodeling.^66^ Transcriptomic profiling of immune-related pathways identified shifts in macrophage populations and T-cell subsets. CIBERSORTx analysis showed an increase in M2 macrophages in human DMD samples and an increase in undifferentiated macrophages in mdx mice. These differences likely reflect species-specific immune dynamics, disease stage, or compensatory mechanisms, such as utrophin up-regulation in mice.^78^ The chronic nature of pathology in older animals may further influence immune cell behavior. Macrophages and T lymphocytes contribute to fibrosis by releasing TGF-β1, which activates fibroblasts and fibroadipogenic progenitors, promoting ECM deposition.^79^^,^^80^ Although M2 macrophages initially support regeneration, prolonged activation in a dystrophic environment rich in IL-4 and IL-13 leads to excessive TGF-β1 secretion and pathologic remodeling.^75^ The Metascape analysis supports this, revealing enrichment in cytokine signaling, myogenesis, and neutrophil degranulation pathways; EMPA's potential influence on macrophage polarization and immune signaling remains an important area for future investigation.

This study acknowledges several limitations. i) Interspecies variation: mouse mdx models have a milder phenotype than human DMD.^81^^,^^82^ Although the DMD^*mdx*^ rat model is closer, it may not fully replicate the disease. Validating findings in a human-relevant model (eg, patient-derived cells, large animal model of DMD) certainly would help to validate these findings. ii) Age differences: human data sets are based on DMD pediatric cohorts, in contrast to the older rodents used, where age-related immune and muscle changes may affect comparisons.^5^ iii) Sample size and treatment duration: larger cohorts and longer treatment may clarify gene-expression trends [eg, serpin family H member 1 (SERPINH1) regulation]. Extending SGLT2i regimens could determine whether sustained treatment enhances immunomodulatory and anti-fibrotic benefits, improving muscle function. iv) Combination therapies: combining SGLT2i with gene therapies or immunomodulatory therapies may yield synergistic benefits. A multi-omics approach should target muscle regeneration, ECM remodeling, and immune dysregulation. Functional assays (eg, treadmill endurance) should directly link molecular changes with clinical outcomes.

## Conclusion

In conclusion, this integrative study combining *in silico* analyses with *in vivo* validation revealed key molecular drivers of ECM remodeling, fibrosis, and immune dysregulation in Duchenne muscular dystrophy. EMPA treatment significantly reduced muscle fibrosis, improved grip endurance, and lowered *Tgfb1* expression in DMD^*mdx*^ rats, highlighting its multi-level therapeutic potential. Although some responses were species specific, the consistent trends across transcriptomic, histologic, and functional data underline the anti-fibrotic and immunomodulatory effects of EMPA. These findings support the repurposing of SGLT2 inhibitors as promising candidates for targeting fibrosis and chronic inflammation in DMD and demonstrate how computational-experimental integration can accelerate drug discovery in rare diseases.

## Disclosure Statement

None declared.

## Supplemental Data

Supplemental Figure S1 Myonuclear localization in DMD and after empagliflozin (EMPA) treatment. **A:** Blue bars indicate the percentage of fibers with centrally located nuclei; red bars indicate the percentage with peripherally located nuclei. DMD shows a marked increase in central nucleation with a reciprocal reduction in peripheral nucleation versus controls. EMPA treatment significantly decreases central nucleation and increases peripheral nucleation relative to DMD, although values remain intermediate between DMD and control. Dots represent individual animals. **B:** C57BL/10ScSnJ (healthy control). **C** and **D:** DMD^*mdx*^ (**C**) and EMPA-treated DMD^*mdx*^ (**D**) mice. Data are given as means ± SEM (**A**). ∗*P* < 0.01, ∗∗∗∗*P* < 0.0001. Scale bar = 20 μm (**B**–**D**).

Supplemental Figure S2 Expression of metabolic transport/uptake genes in a DMD mouse model after empagliflozin (EMPA) treatment. **A**–**D:** Real-time quantitative PCR of glucose and lipid handling markers in C57BL/10ScSnJ (healthy control), DMD^*mdx*^ (DMD), and EMPA-treated DMD^*mdx*^ (EMPA + DMD) mice. **A:***Glut1* is elevated in DMD versus controls and significantly reduced by EMPA (both *P* < 0.01). **B:***Glut4* shows no significant difference among groups. **C:***Cd36* shows no significant difference among groups. **D:***Olr1* is significantly decreased in EMPA-treated mice compared with DMD (*P* < 0.05). Dots represent individual animals. Data are given as means ± SEM (**A**–**D**). ∗*P* < 0.05, ∗∗*P* < 0.01.

Supplemental Figure S3 Cross-study differential expression of metabolic genes in DMD muscle. **A:** Human microarray/RNA-sequencing data sets (GSE1004, GSE3307, GSE38417, and GSE6011). **B:** Mouse data sets (GSE64418, GSE7187, and GSE72151). Each tile shows the log2 fold change (FC; DMD versus control) centered at 0 (red = up, blue = down). Gray tiles marked with × indicate nonsignificant differences after multiple-testing correction [adjusted *P* (padj) ≥ 0.05]. In human cohorts, *CD36* shows data set–specific up-regulation (notably in GSE38417), *PFKFB3* is strongly down-regulated in GSE6011, and *OLR1* is largely unchanged. In mouse cohorts, *Cd36* is decreased in GSE7187, whereas *Pfkfb3* and *Olr1* show predominantly nonsignificant changes. Overall, effects are modest and cohort dependent. Links: *https://www.ncbi.nlm.nih.gov/geo/query/acc.cgi?acc=GSE38417*; accession number GSE38417; *https://www.ncbi.nlm.nih.gov/geo/query/acc.cgi?acc=GSE1004*; accession number GSE1004; *https://www.ncbi.nlm.nih.gov/geo/query/acc.cgi?acc=GSE6011*; accession number GSE6011; *https://www.ncbi.nlm.nih.gov/geo/query/acc.cgi?acc=GSE3307*; accession number GSE3307; *https://www.ncbi.nlm.nih.gov/geo/query/acc.cgi?acc=GSE72151*; accession number GSE72151; *https://www.ncbi.nlm.nih.gov/geo/query/acc.cgi?acc=GSE7187*; accession number GSE7187; and *https://www.ncbi.nlm.nih.gov/geo/query/acc.cgi?acc=GSE64418*; accession number GSE64418.
