# Supplementary figures and images for: Targeting Skeletal Muscle in Duchenne Muscular Dystrophy: Integrating in Silico and Experimental Approaches to Sodium-Glucose Cotransporter-2 Inhibition
Source: Am J Pathol. 2025 Dec 13;196(3):745–65. doi: 10.1016/j.ajpath.2025.11.002 (PMC12975353; doi:10.1016/j.ajpath.2025.11.002)

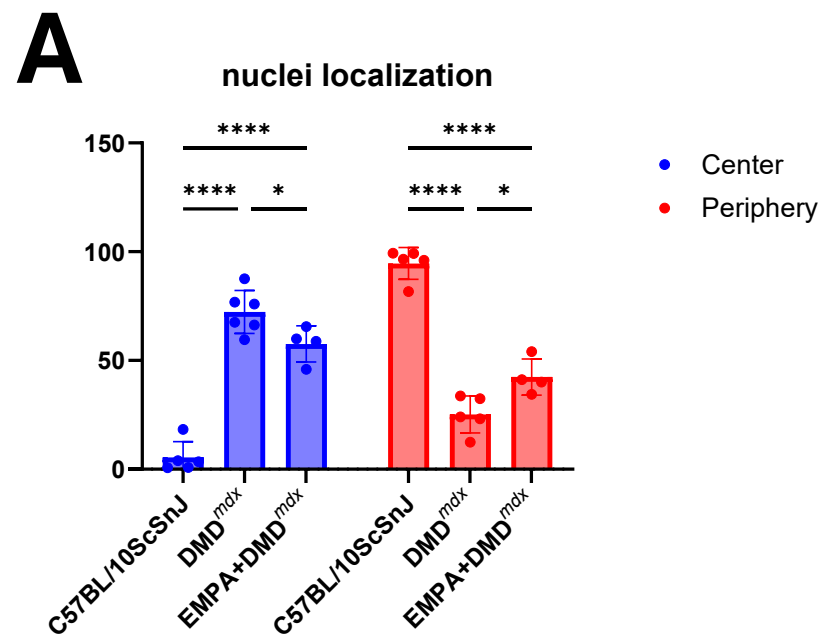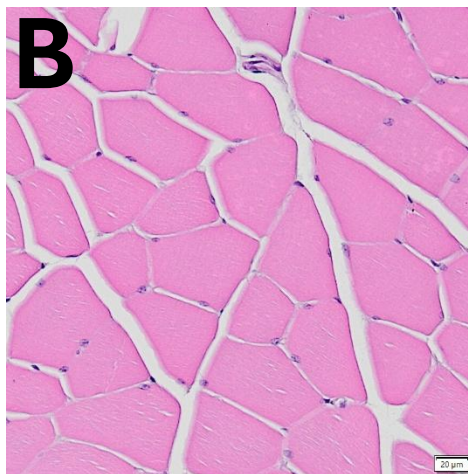

C57BL/10ScSnJ

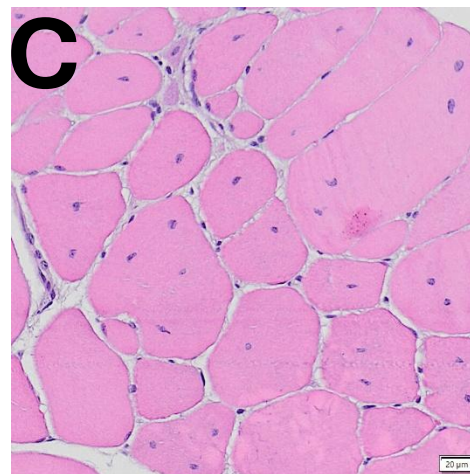

DMD<sup>mdx</sup>

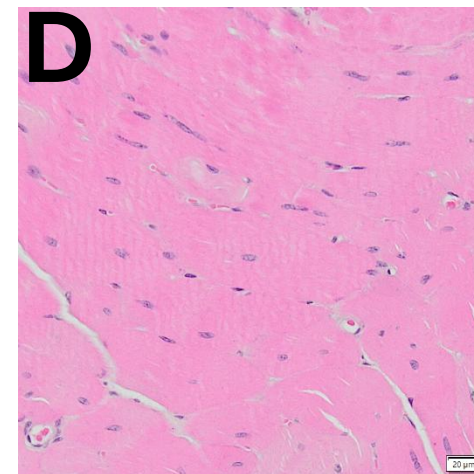

EMPA + DMD<sup>mdx</sup>

Supplement: Supplemental Figure S1 — Myonuclear localization in DMD and after empagliflozin (EMPA) treatment. A: Blue bars indicate the percentage of fibers with centrally located nuclei; red bars indicate the percentage with peripherally located nuclei. DMD shows a marked increase in central nucleation with a reciprocal reduction in peripheral nucleation versus controls. EMPA treatment significantly decreases central nucleation and increases peripheral nucleation relative to DMD, although values remain intermediate between DMD and control. Dots represent individual animals. B: C57BL/10ScSnJ (healthy control). C and D: DMDmdx (C) and EMPA-treated DMDmdx (D) mice. Data are given as means ± SEM (A). ∗P < 0.01, ∗∗∗∗P < 0.0001. Scale bar = 20 μm (B–D). [file mmc1.pdf]

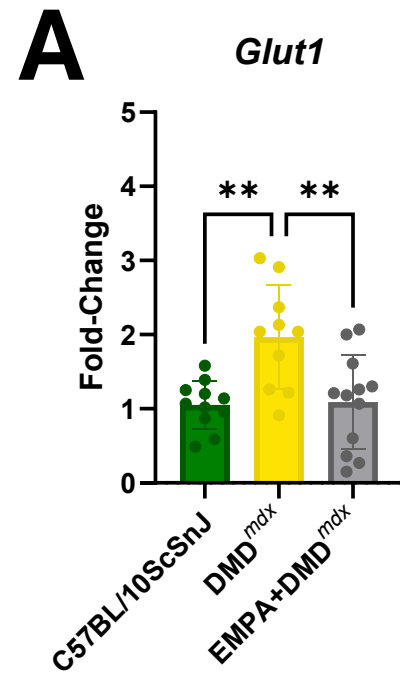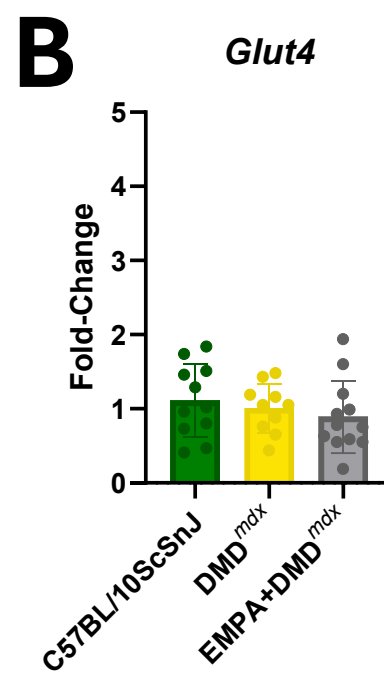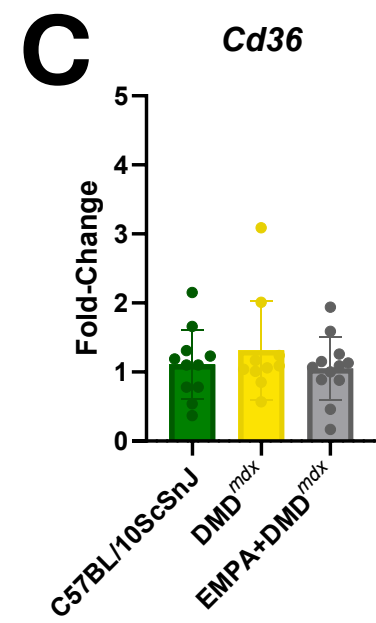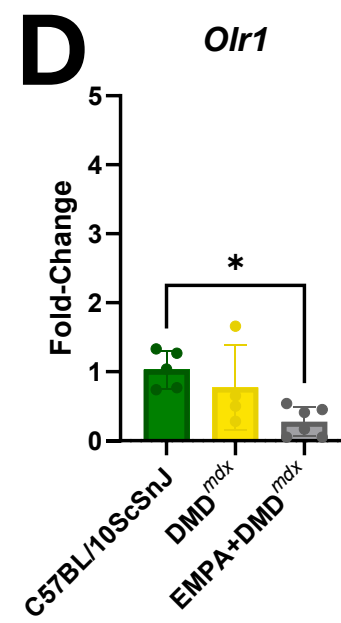

Supplement: Supplemental Figure S2 — Expression of metabolic transport/uptake genes in a DMD mouse model after empagliflozin (EMPA) treatment. A–D: Real-time quantitative PCR of glucose and lipid handling markers in C57BL/10ScSnJ (healthy control), DMDmdx (DMD), and EMPA-treated DMDmdx (EMPA + DMD) mice. A:Glut1 is elevated in DMD versus controls and significantly reduced by EMPA (both P < 0.01). B:Glut4 shows no significant difference among groups. C:Cd36 shows no significant difference among groups. D:Olr1 is significantly decreased in EMPA-treated mice compared with DMD (P < 0.05). Dots represent individual animals. Data are given as means ± SEM (A–D). ∗P < 0.05, ∗∗P < 0.01. [file mmc2.pdf]
