# Supplementary material for: Targeting Skeletal Muscle in Duchenne Muscular Dystrophy: Integrating in Silico and Experimental Approaches to Sodium-Glucose Cotransporter-2 Inhibition
Source: Am J Pathol. 2025 Dec 13;196(3):745–65. doi: 10.1016/j.ajpath.2025.11.002 (PMC12975353; doi:10.1016/j.ajpath.2025.11.002)
Supplement: Supplemental Figure S3 — Cross-study differential expression of metabolic genes in DMD muscle. A: Human microarray/RNA-sequencing data sets (GSE1004, GSE3307, GSE38417, and GSE6011). B: Mouse data sets (GSE64418, GSE7187, and GSE72151). Each tile shows the log2 fold change (FC; DMD versus control) centered at 0 (red = up, blue = down). Gray tiles marked with × indicate nonsignificant differences after multiple-testing correction [adjusted P (padj) ≥ 0.05]. In human cohorts, CD36 shows data set–specific up-regulation (notably in GSE38417), PFKFB3 is strongly down-regulated in GSE6011, and OLR1 is largely unchanged. In mouse cohorts, Cd36 is decreased in GSE7187, whereas Pfkfb3 and Olr1 show predominantly nonsignificant changes. Overall, effects are modest and cohort dependent. Links: https://www.ncbi.nlm.nih.gov/geo/query/acc.cgi?acc=GSE38417; accession number GSE38417; https://www.ncbi.nlm.nih.gov/geo/query/acc.cgi?acc=GSE1004; accession number GSE1004; https://www.ncbi.nlm.nih.gov/geo/query/acc.cgi?acc=GSE6011; accession number GSE6011; https://www.ncbi.nlm.nih.gov/geo/query/acc.cgi?acc=GSE3307; accession number GSE3307; https://www.ncbi.nlm.nih.gov/geo/query/acc.cgi?acc=GSE72151; accession number GSE72151; https://www.ncbi.nlm.nih.gov/geo/query/acc.cgi?acc=GSE7187; accession number GSE7187; and https://www.ncbi.nlm.nih.gov/geo/query/acc.cgi?acc=GSE64418; accession number GSE64418. [file mmc3.pdf]

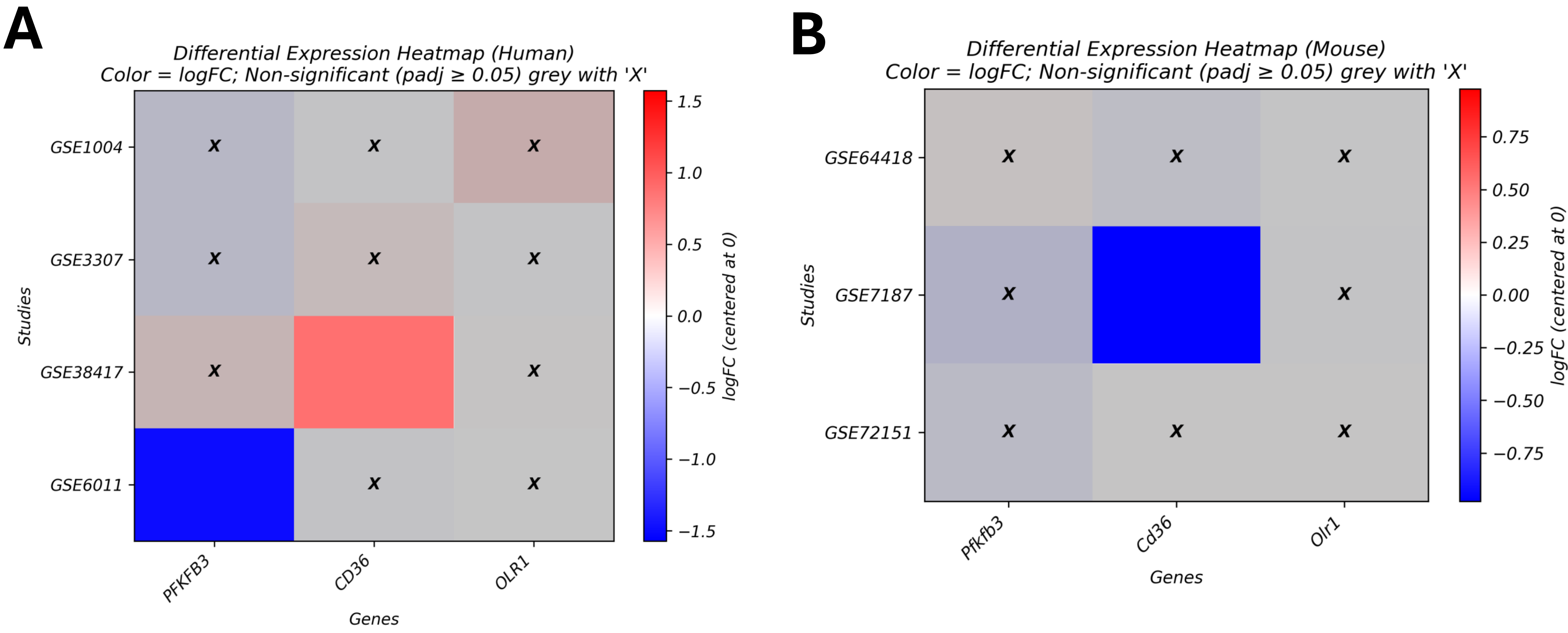
A
Differential Expression Heatmap (Human)
Color = logFC; Non-significant (padj ≥ 0.05) grey with 'X'
GSE1004
GSE3307
GSE38417
GSE6011
Studies
PFKFB3
CD36
OLR1
Genes
logFC (centered at 0)
1.5
1.0
0.5
0.0
−0.5
−1.0
−1.5
B
Differential Expression Heatmap (Mouse)
Color = logFC; Non-significant (padj ≥ 0.05) grey with 'X'
GSE64418
GSE7187
GSE72151
Studies
Pfkfb3
Cd36
Olr1
Genes
logFC (centered at 0)
0.75
0.50
0.25
0.00
−0.25
−0.50
−0.75
